# Combating the Co-Circulation of SARS-CoV-2 and Seasonal Influenza: Identifying Multi-Dimensional Factors Associated with the Uptake of Seasonal Influenza Vaccine among a Chinese National Sample

**DOI:** 10.3390/vaccines12091005

**Published:** 2024-09-01

**Authors:** Xiaoying Zhang, Pinpin Zheng, Xuewei Chen, Ang Li, Lixin Na

**Affiliations:** 1 College of Public Health, Shanghai University of Medicine and Health Sciences, Shanghai 201318, China; zhangxy_21@sumhs.edu.cn (X.Z.); liang@stu.sumhs.edu.cn (A.L.); 2 School of Public Health, Fudan University, Shanghai 200032, China; 3 School of Community Health Sciences, Counseling & Counseling Psychology, Oklahoma State University, Stillwater, OK 74078, USA; xuewei.chen@okstate.edu

**Keywords:** seasonal influenza vaccination, multi-dimensional factors, knowledge, Health Belief Model, cues to action, patient–provider relationship

## Abstract

Introduction: The co-circulation of COVID-19 and seasonal influenza highlighted the importance of promoting influenza vaccination. However, the influenza vaccination rate among the Chinese population is low and requires further promotion. This study examined multi-dimensional factors, such as knowledge of seasonal influenza, health perceptions, cues to action, patient–provider relationships, and COVID-19 pandemic-related factors, in relation to the uptake of the seasonal influenza vaccine (SIV) among the Chinese population. Methods: A cross-sectional, self-administered online survey using a quota sampling method was conducted among Chinese adults 18 years and older between June and August 2022. Multivariate logistic regression was performed to explore factors associated with the 2021 SIV behavior. Results: A total of 3161 individuals from different regions of China were included in this study. The multivariate logistic regression demonstrated that perceived severity of influenza, perceived barriers to taking SIV, cues to action, a stable relationship with providers, worry about contracting COVID-19 in immunization settings, non-pharmaceutical interventions (NPIs), and awareness of the influenza vaccine in protecting against COVID-19 were significantly associated with the SIV uptake. Conclusions: This study examined multi-dimensional factors that may influence SIV uptake. Health promotion programs should incorporate multi-dimensional factors, including personal and environmental factors, related to SIV promotion during the co-circulation period.

## 1. Introduction

Seasonal influenza (abbreviated as influenza) is a respiratory infectious disease caused by influenza viruses that is seriously harmful to human health. People are generally susceptible to influenza viruses. They can infect up to 20% of the world’s population and cause substantial mortality [1]. Seasonal influenza can cause various complications [2] and exacerbate pre-existing health problems [3,4,5]. Seasonal influenza also brings about substantial disease and economic burden. It causes three to five million cases of severe illness and about 290 to 650 thousand respiratory deaths each year on a global basis [1]. In China, there are 84 to 144 million influenza cases every year, causing 96 to 240 thousand deaths each year [6]. In 2019, the influenza-related economic burden in China was RMB 26.3 billion (approximately USD 3.7 billion) [7].

Although the COVID-19 pandemic is no longer considered a global emergency, it is still evolving, as new variants are emerging and reinfection is common [8,9]. The co-circulation of the novel coronavirus SARS-CoV-2 and seasonal influenza will impose significant burdens on the healthcare system [10]. The co-circulation also lends itself to the increased co-infection of influenza and COVID-19, which has been demonstrated to result in more severe clinical outcomes, including rates of mechanical ventilation, ICU admission, as well as odds of death [11,12,13,14]. Furthermore, the COVID-19 pandemic altered the typical patterns of seasonal influenza, with earlier peaks and longer durations in 2022 in the northern hemisphere [15]. Therefore, we are facing more challenges in preventing and controlling seasonal influenza, and it is now more imperative than ever to do so.

Influenza vaccination is the most effective means to prevent influenza and reduce influenza-related severe illness and death [16,17]. The World Health Organization and some country authorities, including China, recommend that individuals over the age of six months who do not have contraindications receive the seasonal influenza vaccine (SIV) on an annual basis [18,19,20]. However, the influenza vaccine coverage rate among Chinese adults was low. A national cross-sectional study reported that the overall influenza vaccination rate in China for the 2018/19 influenza season was 2.4% [21], compared to 45.3% in the U.S. [22]. Another earlier urban telephone survey reported that the vaccination rates in the 2009/10 season, 2010/11 season, and 2011/12 season were 9.4%, 11.3%, and 6.4%, respectively [23]. The low vaccination rates indicate that the vast majority of the Chinese population was not vaccinated. Consequently, it is imperative to enhance the influenza vaccination rate among the Chinese population.

Understanding factors of SIV uptake is essential for designing effective interventions to advocate vaccination behavior. The previous literature has reported on factors associated with SIV uptake, such as perceived vaccine efficacy and disease severity, healthcare providers’ recommendations, free vaccination policy, and the history of influenza vaccination [24,25]. However, other factors, such as cues to action factors other than healthcare providers’ recommendations (e.g., recommendations from family and friends) as well as patient–provider relationships, were not thoroughly examined. Moreover, as the majority of studies were conducted before the COVID-19 pandemic, the findings are unable to provide recommendations for the promotion of SIV during the period of co-circulation. This study sought to examine multidimensional factors of Chinese people’s influenza vaccination behavior, including knowledge about seasonal influenza, health perceptions, cues to action, patient–provider relationships, and factors related to the COVID-19 pandemic.

The first dimension is knowledge about seasonal influenza and influenza vaccine, as the previous literature suggests its significance in individuals’ vaccination behavior [26,27,28]. Health perception is the second dimension. According to the Health Belief Model [29], and as suggested by previous literature [30,31,32,33], this study examined four aspects of health perceptions: perceived susceptibility, perceived severity, perceived benefits, and perceived barriers. Furthermore, this study explores the potential effects of cues to action factors, which represent another construct in the Health Belief Model [29]. Cues to action refers to the stimuli that trigger the decision-making process to take a recommended health action, which can be internal or external [29]. As mentioned earlier, the literature merely focuses on healthcare providers’ recommendations [34,35], leaving other potential cues to action factors, such as recommendations from family and friends and community health education, understudied [36,37]. This current research explores the effects of the “cues to action” factors in a more comprehensive manner.

The patient–provider relationship is of paramount importance in healthcare and medical practice. Previous literature reported on the influence of the patient–provider relationship on vaccination [38,39,40]. However, to the knowledge of the authors, no study has yet explored the effects of the patient–provider relationship on influenza vaccination intention or behavior. This study aims to explore the correlation between patient–provider relationship and SIV uptake behavior. Three aspects of the patient–provider relationship were examined: whether individuals have a regular or consistent healthcare provider, their relationship with their provider, and their level of trust in their providers. These three aspects were selected for their pivotal role in the patient–provider relationship in the Chinese context.

The last dimension comprises the COVID-19 pandemic-related factors. This study was conducted during the pandemic (June to August 2022) and examined the association between pandemic-related factors and SIV. Three COVID-19 pandemic-related factors were examined in this study. First, the possible influence of non-pharmaceutical solutions (NPIs), such as mask-wearing, social distancing, and handwashing, on seasonal influenza vaccination was examined. Secondly, this study examined whether concerns about contracting COVID-19 at the SIV immunization site were negatively associated with SIV uptake behavior. Finally, we examined whether individuals’ awareness of the protective effect of seasonal influenza vaccination against COVID-19 infection was associated with their uptake of SIV. Better knowledge of the influence of COVID-19 pandemic-related factors helps us enhance the influenza vaccination rate during the co-circulation stage.

**Figure 1:**
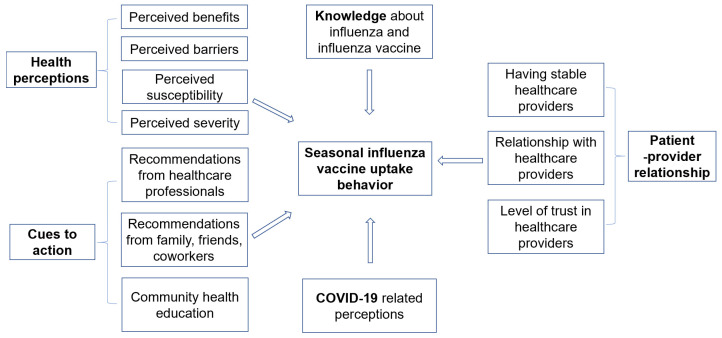
Multi-dimensional factors associated with seasonal influenza vaccination uptake.

In light of the co-circulation of SARS-CoV-2 and seasonal influenza, it is imperative to enhance the influenza vaccination rate among the Chinese population. The objective of this study is to analyze the multi-dimensional factors associated with individuals’ SIV uptake. Figure 1 presents the multi-dimensional factors included in this study. The majority of these factors have not been previously investigated in depth. The findings of the study will help us better understand the factors correlated with people’s influenza vaccination behavior, which will guide the development of tailored programs to improve influenza vaccine coverage in China.

## 2. Materials and Methods

### 2.1. Study Design and Participants

A cross-sectional, self-administered online survey was conducted among Chinese adults aged 18 and above from June to August 2022 via Wenjuanxing (Changsha Ranxing Information Technology Co., Ltd., Changsha, China). Wenjuanxing is the largest commercial survey design platform and survey sampling and administration company in China, which recruits individuals who sign up on the website to take paid surveys across multiple sites nationally. To ensure a more representative sample, we adopted a region-based quota sampling method, where the proportions of participants from the northeast, eastern, central, and western regions were similar to those in the census. Eligibility criteria for the online survey included Chinese individuals aged 18 years and older and residing on the Chinese mainland. Wenjuanxing sent emails to eligible individuals who had previously signed up to receive online survey invitations. The email offered compensation worth CNY 6 Chinese (approximately USD 1) for each respondent. Prior to answering the questions, informed consent was recorded electronically. Ethical approval was obtained from the ethical review board of Shanghai University of Medicine and Health Sciences.

### 2.2. Questionnaire Development and Measure

All survey questions were initially adopted from national surveys and the related literature. The survey questions were then reviewed and revised by an expert review panel consisting of epidemiologists and behavior scientists. The updated survey was pilot-tested among 20 people to collect comments on the appropriateness of the content, language use, literacy level, and logic settings among the questions. The questionnaire included the following sections: (1) socio-demographic variables; (2) pre-existing diseases; (3) knowledge about seasonal influenza and influenza vaccine; (4) perceived susceptibility and perceived severity of influenza, and perceived benefits of and barriers to vaccination; (5) cues to action for taking up SIV (e.g., the recommendation from healthcare providers); (6) patient–provider relationship; and (7) COVID-19 pandemic-related factors. All questions were closed-ended, with tick boxes provided for responses.

#### 2.2.1. Background Factors

Background factors included age, gender, education level, employment status, regions (eastern, central, western, northeast), community type (rural or urban), medical background (whether or not the individual received medical training), and chronic disease status.

#### 2.2.2. Vaccination Behavior

Vaccination behavior was assessed by a single question: “Have you taken up the seasonal influenza vaccine since 1 January 2021?” People could choose from a Likert scale from “definitely not” (=1) to “definitely yes” (=4). As in other studies [41,42], a binary variable was created by recoding responses 3 and 4 (affirmative) as “1” and all other responses as “0” (non-affirmative).

#### 2.2.3. Knowledge about Influenza and Influenza Vaccine

The knowledge of influenza and influenza vaccine was assessed using seven multiple-choice questions adopted from the previous literature [26,27,28]. The example question was, “People with underlying diseases are among the priority groups to take the influenza vaccine [disagree/agree/don’t know]”. Responses were coded as 1 for accurate perceptions and 0 for erroneous perceptions, including the response “don’t know”. The theoretical range for the knowledge sum score was from 0 to 7.

#### 2.2.4. Perceived Benefits and Barriers of Taking Vaccine

The perceived benefits of the influenza vaccination were assessed by four items: (1) influenza vaccine is effective; (2) influenza vaccine can decrease the chance of contracting influenza; (3) influenza vaccine can eliminate the severity of influenza infection; and (4) getting vaccinated for me is also a protection for children and older adults at home. These items were developed from the Health Belief Model [29] and adopted from previous works [43,44,45]. Each question was evaluated on a four-point Likert scale, with responses ranging from 1 (disagree) to 4 (agree), and the sum score ranged from 4 to 16. Five questions were constructed to assess participants’ perceived barriers to seasonal influenza vaccination based on its definition and the previous literature [46,47,48]. The five aspects identified as potential barriers to seasonal influenza vaccination are lack of knowledge about the influenza vaccine, concerns about the cost (perceived as too expensive), concerns about potential side effects, limited access to vaccination venues, and insufficient supply of the vaccine. Each question was scored on a four-point Likert scale, with responses ranging from 1 (not a barrier) to 4 (a significant barrier). The sum score ranged from 5 to 20.

#### 2.2.5. Perceived Susceptibility and Severity of Influenza

Both perceived susceptibility and perceived severity were assessed by two questions adopted from previous literature [43,44,45]. The two items for perceived susceptibility were the perceived probability of acquiring influenza and the perceived probability of developing severe symptoms of seasonal influenza. The two items assessing perceived severity were the severity of influenza perceived by the individual and their family. All four items were scored on a four-point Likert scale, with 1 representing “unlikely”, 2 representing “somewhat unlikely”, 3 representing “somewhat likely”, and 4 representing “very likely”. The sum score for each variable ranged from 2 to 8.

#### 2.2.6. Cues to Action for Influenza Vaccination

Three aspects of cues to action were measured: healthcare providers’ recommendations, family and friends’ recommendations, and community health education. These three questions were developed by the research team based on its definition and previous literature [25,29,34,49]. An example question was, “Do your providers recommend you vaccinate against influenza?” Participants can choose from “never” (1) to “always” (4).

#### 2.2.7. Patient–Provider Relationships

The research team constructed three questions to measure patient–provider relationships with consideration of the Chinese context. The first question is about whether people have a regular source of healthcare. Respondents may indicate that they do not have a regular provider (coded as 1) to that they have regular healthcare providers (coded as 4). This question was constructed in light of the fact that China has trialed family doctor contract services in some cities, which enables the building of stable patient–provider relationships [50,51]. The remaining two aspects pertain to the relationship between individuals and healthcare providers, as well as the level of trust individuals place in these providers. Each of these aspects was evaluated on a four-point Likert scale. A higher score indicated a better relationship with and a higher level of trust in the healthcare provider.

#### 2.2.8. COVID-19 Related Factors

Three questions were constructed to assess the association between COVID-19 pandemic-related factors and SIV uptake. The questions were as follows: (1) whether the influenza vaccine can help prevent COVID-19 infection (yes/no), (2) whether the COVID-19 pandemic prevents them from taking up the influenza vaccine because of concerns about contracting COVID-19 at immunization sites) (yes/no), and (3) whether NPIs decrease their intention to vaccinate against influenza (yes/no).

The investigation lasted for two months. Responses were excluded if the total answering time was less than three minutes, as a shorter answering time negatively affects the quality of the responses based on the length of the questionnaire and the average reading speed of the Chinese. A total of 3170 responses met the criteria. Nine respondents reported being younger than 18 years old and thus excluded from the analysis, so 3161 participants were included in the analysis.

### 2.3. Statistical Analysis

Stata 17.0 (Stata Corp, College Station, TX, USA) was used for data management and analysis. Data cleaning was performed before the analysis. Descriptive statistics were performed to describe the participants’ socio-demographic characteristics, knowledge about influenza and influenza vaccine, health beliefs about SIV, cues to action for vaccination, patient–provider relationships, and COVID-19-related factors. Bivariate analysis was conducted to assess the association between socio-demographic characteristics and other independent variables and individuals’ influenza vaccination behavior. Only those background variables with a significant correlation with vaccination were included in the model as covariates. A multivariate logistic regression model was performed to examine the factors associated with influenza vaccine uptake, and the OR and 95% CIs were used as measures of association. All statistical tests were two-sided, with *p* < 0.05 considered statistically significant.

## 3. Results

### 3.1. Characteristics of Study Participants

A total of 3161 individuals from 30 provinces, municipalities and autonomous regions were included in the present study. The characteristics of the participants are presented in Table 1.

The majority of participants (89.9%) were employed, 78.9% resided in urban areas, 67.0% had attained a bachelor’s or higher degree, and 58.2% were female. Furthermore, 17.9% of the participants had chronic diseases, and 15.4% had a medical background. The age distribution of the participants was as follows: 18–30 (43.9%), 31–45 (26.7%), and >46 (29.4%). The regional distribution of the sample population was eastern (40.2%), central (24.6%), western (25.7%), and northeast (9.5%), which is comparable to the national population distribution from the seventh census data [52]. The income distribution was as follows: RMB < 6000 (41.8%), 6000–8000 (38.0%), and >10,000 (20.3%).

Table 2 presents the descriptive statistics of the key independent variables. The average score of the knowledge scale was 5.26 (0 to 7, SD = 1.46). The mean score of the perceived benefits scale was 12.70 (SD = 1.72), ranging between 4 and 16. The mean score of perceived barriers was 11.75 (SD = 1.90), ranging from 5 to 20. The total scores for perceived susceptibility and perceived severity ranged from 2 to 8, with a mean of 3.64 (SD = 1.40) and 5.11 (SD = 1.52), respectively.

The scores for the “cues to action” items all ranged from 1 to 4. The average scores were 2.53 (SD = 0.96), 2.39 (SD = 0.96), and 2.45 (SD = 0.79), respectively, for recommendations from healthcare providers, recommendations from family, friends and workmates, and community health education. The scores for the three aspects of patient–provider relationship also ranged from 1 to 4. The mean scores were 2.20 (SD = 0.89), 2.30 (SD = 0.85), and 3.15 (SD = 0.54) for whether the individual has a stable provider, the relationship with the provider, and the level of trust in the provider. Only 32.9% (n = 1039) of the participants have a stable or relatively stable healthcare provider. Less than half of the participants (43.8%) were found to be familiar with their healthcare providers. Nevertheless, the majority of the participants (93.4%) demonstrated a high level of trust in their healthcare providers. Moreover, over half of the respondents perceived COVID-19 as a barrier to receiving the influenza vaccine (55.6%), believed that the influenza vaccine could prevent COVID-19 infection (56.7%), and reported that NPIs had decreased their intention to receive the influenza vaccine (50.6%).

### 3.2. Background Factors of Vaccination Uptake

The overall influenza vaccination rate in the year 2021 was 35.27%. Table 3 presents the associations between background factors and influenza vaccine uptake behavior. Individuals with a bachelor’s or higher education level were more likely to be vaccinated (OR = 1.81, 95% CI: 1.53, 2.13), as were those with a medical background (OR = 2.09, 95% CI = 1.72, 2.54). The results indicate that individuals with higher incomes (<6000 = 1, 6000~8000: OR = 1.52, 95% CI = 1.24, 1.85; >10,000: OR = 2.03, 95% CI: 1.61, 2.57) and those with chronic diseases (OR = 1.49, 95% CI = 1.24, 1.80) were more likely to be vaccinated. Factors negatively associated with vaccine uptake included being older than 46 years (OR = 0.48, 95% CI = 0.40, 0.58) and being unemployed (OR = 0.74, 95% CI = 0.58, 0.95).

### 3.3. Factors Associated with SIV Uptake Behavior

To assess the presence of multicollinearity among the predictor variables in the multivariate logistic regression model, we calculated the Variance Inflation Factor (VIF) for each variable. All VIF values were found to be well below the commonly accepted threshold of 5 (ranged from 1.02 to 2.10), indicating that multicollinearity is not a concern in this model [53]. Table 4 presents the univariate logistic regression (cOR) and multivariate logistic regression (aOR) results for vaccination uptake behavior. In the univariate logistic regression results, knowledge (cOR = 1.23, 95% CI: 1.16, 1.29), higher perceived benefits (cOR = 1.25, 95% CI: 1.19, 1.31), higher perceived susceptibility (cOR = 1.17, 95% CI: 1.11, 1.23), and higher perceived severity (cOR = 1.35, 95% CI: 1.28, 1.42) were significantly associated with higher odds of taking vaccination. In addition, people who received more frequent recommendations from providers (cOR = 2.84, 95% CI: 2.58, 3.12) and from family and friends (cOR = 3.29, 95% CI: 2.97, 3.64), and who received more frequent community health education on vaccination (cOR = 3.07, 95% CI: 2.74, 3.43), had higher odds of taking the influenza vaccine. Furthermore, people with stable healthcare providers (cOR = 2.33, 95% CI: 2.00, 2.72), keeping good relations with providers (cOR = 2.16, 95% CI: 1.86, 2.50), and having a higher level of trust in providers (cOR = 2.12, 95% CI: 1.51, 2.98) had higher odds of taking the influenza vaccine. Last, the belief that the influenza vaccine can help prevent COVID-19 infection (cOR = 4.31, 95% CI: 3.65, 5.09) was also related to higher vaccination odds. On the other hand, factors negatively associated with vaccination included perceiving higher barriers to taking the vaccine (cOR = 0.81, 95% CI: 0.78, 0.84), worrying about contracting COVID-19 when going out to take the influenza vaccine (cOR = 0.88, 95%CI: 0.81, 0.95), and perceiving that NPIs decreased their intention to take the flu vaccine (cOR = 0.59, 95% CI: 0.51, 0.68).

The multivariate logistic regression was adjusted for background variables. Among the four health perceptions from the Health Belief Model, perceived barriers to taking SIV (aOR = 0.90, 95% CI: 0.86, 0.95) and perceived severity of influenza infection (aOR = 1.12, 95% CI: 1.05, 1.20) were significantly associated with vaccination uptake. All three aspects of “cues to action” were significantly associated with vaccination uptake, among which the odds ratio for recommendations from healthcare providers, recommendations from family and friends, and community health education were 1.38 (95% CI: 1.21, 1.58), 1.97 (95% CI: 1.73, 2.25) and 1.44 (95% CI: 1.24, 1.67), respectively. For the three items of patient–provider relation, only having a regular healthcare provider was significantly associated with increased odds of taking influenza vaccine (aOR = 1.37, 95% CI: 1.07, 1.76). For COVID-19-related factors, we found that compared to those who did not perceive the COVID-19 pandemic as a barrier for them to go out to take up the influenza vaccine, those who reported the pandemic as a barrier were 0.84 time less likely to take the 2021 influenza vaccine (aOR = 0.84, 95% CI: 0.76, 0.93). Compared to people who did not believe that influenza vaccination would help prevent COVID-19 infection, those who believed so were 2.51 times more likely to receive the 2021 influenza vaccine (aOR = 2.51, 95% CI: 2.07, 3.05). Lastly, people who disclosed that the NPIs (washing hands, wearing a mask, keeping social distance, etc.) decreased their intention to take the flu vaccine were 0.65 times less likely to receive the 2021 influenza vaccine than those who did not disclose that (aOR = 0.65, 95% CI: 0.54, 0.79).

## 4. Discussion

We examined the associations of knowledge about influenza and influenza vaccines, health perceptions, cues to action factors, patient–provider relationship, and COVID-19 pandemic-related factors with seasonal influenza vaccination behavior among the Chinese population. The influenza vaccination coverage rate for our sample in the year 2021 was 35.27%, which is higher than the previously reported SIV coverage rate in China [54]. The higher vaccination rate may be due to the higher education level of the study sample, as education level was positively associated with the influenza vaccination rate [55]. Despite the higher vaccination rate here than in previous studies, the general influenza vaccination coverage is lower than that of other countries such as the US (50.2% among adults ≥ 18 years for 2020–2021 flu season) [56] and Canada (40% among adults ≥ 18 years for 2020–2021 flu season) [57]. Efforts are needed to enhance the SIV coverage among the Chinese population.

We found that older age, higher income, pre-existing diseases, perceived severity of influenza, cues to action factors, having a stable healthcare provider, and COVID-19-related factors and perceptions were significantly associated with SIV uptake among Chinese people. Interestingly, this study exhibited that middle-aged and older people (>45 years) had lower influenza vaccination rates than young people (18–30 years). Previous studies reported that older adults (>60 years) had a higher vaccination rate [54], but little attention has been paid to the middle-aged population. Several reasons might contribute to the low vaccination rate among the middle-aged and older population. To begin with, given the low influenza vaccination coverage rate in Chinese society, the influenza vaccine is still “new” to some people. Young people are usually more open and receptive to new things than their middle-aged or older counterparts, which might contribute to their higher vaccination rate. Furthermore, young people may also receive more health education on influenza vaccines than middle-aged and older people, since health education increasingly employs mHealth or eHealth methods that reach young people more effectively [58,59]. In addition, there may be a specific reason for the low vaccination rate among the middle-aged population. Unlike older adults, who are among the prioritized populations to receive the influenza vaccine, middle-aged people may be the “overlooked” population in vaccination promotion. Future studies should investigate the factors influencing vaccination acceptance among the middle-aged and older population. This finding has significant implications for practice. In addition to older adults, the middle-aged population should also be targeted for SIV promotion, given that they are entering their elderly years in the coming years. Consequently, novel health education strategies are warranted to publicize the benefits and necessity of the influenza vaccine for the middle-aged and older population.

Further, we found that people with underlying medical conditions were 1.49 times more likely to receive the influenza vaccine (OR = 1.49), which is consistent with the previous literature [25,31]. It is plausible that people with underlying diseases may receive recommendations from healthcare providers to take up SIV. Besides this, higher income was associated with higher odds of receiving influenza vaccination (OR = 1.34), which is expected since influenza vaccination is not covered by the National Immunization Program, and most people in China pay out-of-pocket for this vaccine. In some regions of China, the influenza vaccine has been provided free of charge to individuals aged sixty years and older, which has led to a notable increase in the vaccination uptake rate [60]. Making influenza vaccines free or covered by insurance for some priority groups might be considered.

Among the four health perceptions in the Health Belief Model, perceived severity and perceived barriers were found to be significantly associated with the uptake of SIV in the multivariate logistic regression. With regard to perceived severity, a considerable proportion of the Chinese population is under the impression that influenza is not a severe illness [61]. This myth may be attributed to the translation of influenza into “epidemic cold” in Chinese, which emphasizes the susceptibility of influenza, but not its severity. In the knowledge scale of this survey, 20% of the participants indicated that they were unsure or disagreed with the statement that influenza can cause severe complications. As people’s knowledge about influenza and influenza vaccination is an important component of vaccine literacy [62], our finding indicates the necessity of increasing people’s vaccine literacy, as the previous literature indicated the positive association between health literacy and vaccination intention [63,64]. Health education should focus on communicating the seriousness and consequences of contracting influenza. Moreover, perceived barriers were also found to be significantly associated with the uptake of SIV. This study examined five aspects of barriers to SIV, namely, price, poor access, short storage, concerns about side effects, and lack of knowledge about SIV. This finding indicates that numerous barriers remain to SIV among Chinese individuals. Significant efforts are required to address these obstacles and enhance SIV uptake.

For “cues to action” factors, all three items are significantly associated with vaccination behavior. This implies that compared to individuals’ health perceptions, cues to action factors might be of greater importance in Chinese people’s vaccination behavior. This may be particularly pertinent in the context of exceptional circumstances, such as the COVID-19 pandemic, where individuals may need additional guidance in health-related behaviors. We found that recommendations from healthcare providers were significantly positively associated with their SIV uptake behavior in the past year, which is consistent with the findings of previous studies [31,32,54]. Further research is required to identify the factors that facilitate or impede healthcare providers’ recommendations for the uptake of SIV. Furthermore, it is recommended that healthcare providers receive additional training and guidance to enhance their ability to provide more effective recommendations. For example, the US Centers for Disease Control and Prevention (CDC) recommends that healthcare providers utilize the “SHARE” approach to make recommendations for patients to receive the influenza vaccine [65]. This approach involves sharing reasons for vaccination, highlighting positive experiences, addressing patient questions, reminding patients that influenza vaccines help protect them and their loved ones, and explaining the potential costs of getting influenza.

Recommendations from family and friends also played a critical role in influenza vaccine uptake, which is supported by previous literature [37]. The Diffusion of Innovation Theory states that innovations, such as new ideas, behaviors, technologies, or goods, spread through a population gradually rather than all at once [66]. Influenza vaccination as a behavior can be regarded as an innovation for some people, given the low SIV coverage rate among the general population in China. In front of innovation, there are five types of adopters: innovators, early adopters, early majority, late majority, and laggards. The adoption of the new behavior starts with innovators and early adopters, and then spreads through the population to the early majority and late majority. Based on the finding that recommendations from family and friends played a critical role in influenza vaccine uptake, we can identify innovators and early adopters of influenza vaccination in the community, train them to broadcast the vaccine, and let them mobilize other people in the community to uptake the vaccine. Last but not least, community health education was also essential in increasing influenza vaccine uptake. Novel community health education campaigns should be developed. For instance, a randomized controlled trial found that the video-led educational intervention showed effects on increased uptake of influenza vaccine among older adults in western China [67]. In summary, to boost the influenza vaccination rate, we should incorporate the joint efforts of healthcare providers, people in the community, and innovative community health education.

Among the three items we constructed to measure the patient–provider relationship, only “having a regular provider” was significantly associated with vaccination uptake behavior (aOR = 1.37, 95% CI: 1.07, 1.76) in the multivariate logistic regression. This finding highlights the potential role of a stable patient–provider relationship in increasing the SIV coverage among the general population. It is worth noting that only 33% of the participants reported having stable or relatively stable providers. China has trialed the family doctor contract policy in some cities [50,51], which facilitates establishing stable relationships between patients and providers, providing new opportunities for promoting influenza vaccination. This study also examined the potential influence of the COVID-19 pandemic on influenza vaccine uptake. More than half of the participants reported that the NPIs against COVID-19 decreased their intention to take the influenza vaccine, which was also significantly associated with not taking it in the previous year. We also found that an awareness of the protective effect of flu vaccine for COVID-19 was significantly associated with higher odds of taking SIV in 2021. These findings highlight the significance of health education in relation to the benefits and necessity of influenza vaccine uptake in the face of new situations, not limited to the COVID-19 pandemic, because people needs more guidance about health behavior in new situations. In addition, we found that more than half (55.6%) of the participants expressed concerns about contracting COVID-19 at the vaccination venue, which emerged as a significant barrier to SIV uptake in the year 2021. This finding is supported by the literature [68] and it highlights the significance of enhancing safe access to influenza vaccination during the pandemic. For instance, the WHO recommend additional/alternative locations to increase vaccination coverage [69]. It also recommended that influenza vaccination sessions should be conducted in well-ventilated areas [69].

This study has several limitations to mention. First, because of the cross-sectional study design, causal relationships cannot be established. Longitudinal studies are warranted to confirm the findings of this study. Second, although the sampling was based on the population quota in each economic region, it is still convenience sampling, which may result in selection bias. For instance, the sample is biased toward the highly educated population, which may contribute to the higher SIV uptake rate. Furthermore, this is an online survey, so people who do not have access to the internet or are unfamiliar with the online survey were not represented, and the sample might overrepresent certain demographic groups (e.g., younger, more tech-savvy individuals). Moreover, people who chose to participate in this survey might be those with a particular interest in seasonal influenza vaccine. For instance, people with higher vaccine literacy might be more likely to participate. This self-selection can lead to biased results, such as the higher vaccination rate in this sample. Additionally, our findings may not be generalizable to countries outside of China, as vaccine uptake rates, policies, and delivery systems can vary significantly in other regions. Therefore, caution should be taken in generalizing the results. Fourth, influenza vaccine uptake was self-reported, which could lead to recall bias and socially desirable responses. Fifth, other psychosocial factors, such as self-efficacy regarding SIV uptake, were not assessed in this study.

## 5. Conclusions

This study incorporated multi-dimensional factors to explore both personal (knowledge, perceptions) and environmental (cues to action, patient–provider relationship, COVID-19 pandemic) factors that may influence vaccination behavior during the pandemic. We found that perceived severity, perceived barriers, cues to action factors, stable patient–provider relationship, and COVID-19 pandemic-related factors were the most salient factors affecting people’s influenza vaccination behavior during the pandemic. Future longitudinal and intervention studies are needed to verify the above findings. Multi-dimensional interventions should be implemented to improve influenza vaccine coverage. Health education is needed to eliminate the myths and emphasize the necessity and benefits of SIV during the pandemic and periods with the co-circulation of COVID-19 and influenza. In addition, healthcare providers and community forces should be mobilized to increase the seasonal influenza vaccination rate.

## Figures and Tables

**Table 1:** Descriptive statistics of the background variables (n = 3161).

Characteristics	Numbers/Mean	Percentage/SD
Age		
18–30	1389	43.9%
31–45	844	26.7%
>46	928	29.4%
Gender		
Male	1323	41.9%
Female	1838	58.2%
Education		
College and lower	1042	33.0%
Bachelor or higher	2119	67.0%
Economic region		
Eastern	1269	40.2%
Central	779	24.6%
Western	812	25.7%
Northeast	301	9.5%
Employment status		
Employed	2841	89.9%
Unemployed	320	10.1%
Medical background		
Yes	488	15.4%
No	2673	84.6%
Personal monthly income (RMB)		
<6000 (About USD 840)	1320	41.8%
6000~8000 (USD 840–1120)	1200	38.0%
>10,000 (USD 1400)	641	20.3%
Community type		
Urban	2495	78.9%
Rural	666	21.1%
Chronic diseases		
No disease	2594	82.1%
With diseases	567	17.9%

**Table 2:** Descriptive statistics of the key independent variables (n = 3161).

Predictors	Range	Numbers (%) /Mean (SD)	Cronbach’s Alpha
Knowledge	0~7	5.26 (1.46)	0.49
Influenza is highly contagious	0/1		
Influenza can cause serious complications	0/1		
It is particularly important for people with underlying diseases to receive flu vaccine	0/1		
It is particularly important for children, pregnant women, and older people to receive flu vaccine	0/1		
Seasonal flu vaccine is the best way to prevent influenza and complications	0/1		
Flu vaccine is safe	0/1		
Taking a flu vaccine might cause flu	0/1		
Perceptions			
Perceived benefits	4–16	12.70 (1.72)	0.69
Perceived barriers	5–20	11.75 (1.90)	0.58
Perceived susceptibility	2–8	3.64 (1.40)	0.73
Perceived severity	2–8	5.11 (1.52)	0.85
Cues to action			
Recommendation from healthcare providers	1–4	2.53 (0.96)	
Recommendation from family, friends, workmates	1–4	2.39 (0.96)	
Community health education	1–4	2.45 (0.79)	
Patient–provider relations			
Stable provider	0/1	1039 (32.9%)	
Relationship with provider	0/1	1385 (43.8%)	
Level of trust	0/1	2953 (93.4%)	
COVID-19-related factors			
Perceiving COVID-19 as a barrier for influenza vaccination	0/1	1757 (55.6%)	
Perception that flu vaccine can help prevent COVID-19 infection	0/1	1791 (56.7%)	
Perception that non-pharmaceutical solutions decrease the intention of taking flu vaccine	0/1	1598 (50.6%)	

**Table 3:** The associations between background factors and vaccination behaviors (n = 3161).

Characteristics	OR (95% CI)	*p* Value
Age		
18–30	1	
31–45	0.89 (0.75, 1.06)	0.21
>46	0.48 (0.40, 0.58)	0.00
Gender		
Male	1	
Female	0.98 (0.85, 1.14)	0.80
Education		
College and lower	1	
Bachelor or higher	1.81 (1.53, 2.13)	0.00
Economic regions		
Eastern	1	
Central	1.04 (0.87, 1.26)	0.65
Western	0.98 (0.81, 1.18)	0.82
Northeast	0.87 (0.66, 1.13)	0.29
Employment status		
Employed	1	
Unemployed	0.74 (0.58, 0.95)	0.02
Medical background		
No	1	
Yes	2.09 (1.72, 2.54)	0.00
Personal monthly income		
<6000	1	
6000~8000	1.52 (1.24, 1.85)	0.00
>10,000	2.03 (1.61, 2.57)	0.00
Community type		
Urban	1	
Rural	0.91 (0.76, 1.09)	0.32
Chronic diseases		
No disease	1	
With diseases	1.49 (1.24, 1.80)	0.00

**Table 4:** Factors of influenza vaccine uptake behavior (n = 3161).

Predictors	cOR (95% C.I.)	aOR (95% C.I.)
Knowledge	1.23 (1.16, 1.29) ***	1.01 (0.94, 1.08)
Perceptions		
Perceived benefits	1.25 (1.19, 1.31) ***	0.98 (0.92, 1.04)
Perceived barriers	0.81 (0.78, 0.84) ***	0.90 (0.86, 0.95) ***
Perceived susceptibility	1.17 (1.11, 1.23) ***	1.00 (0.93, 1.07)
Perceived severity	1.35 (1.28, 1.42) ***	1.12 (1.05, 1.20) ***
Cues to action		
Recommendation from healthcare providers	2.84 (2.58, 3.12) ***	1.38 (1.21, 1.58) ***
Recommendation from family, friends, workmates	3.29 (2.97, 3.64) ***	1.97 (1.73, 2.25) ***
Community health education	3.07 (2.74, 3.43) ***	1.44 (1.24, 1.67) ***
Patient–provider relations		
Stable providers	2.33 (2.00, 2.72) ***	1.37 (1.07, 1.76) **
Relationship with providers	2.16 (1.86, 2.50) ***	0.89 (0.70, 1.14)
Level of trust in providers	2.12 (1.51, 2.98) ***	0.73 (0.49, 1.10)
COVID-19-related factors		
Perceiving COVID-19 as a barrier	0.88 (0.81, 0.95) **	0.84 (0.76, 0.93) ***
Perception that flu vaccine help prevent COVID-19 infection	4.31 (3.65, 5.09) ***	2.51 (2.07, 3.05) ***
Perception that NPIs decrease the intention of taking flu vaccine	0.59 (0.51, 0.68) ***	0.65 (0.54, 0.79) ***

Notes: cOR, crude odds ration; aOR, adjusted odds ratio; CI, confidence interval; **, *p* < 0.01; ***, *p* < 0.001. The model was adjusted for background factors, including age, education level, employment status, medical background, personal monthly income, and underlying diseases.

## Data Availability

The data presented in this study are available on request from the corresponding author.
